# Nitrous Oxid Anesthesia

**Published:** 1904-02-15

**Authors:** F. M. Richardson


### NITROUS OXID ANESTHESIA.

BY DR. F. M. RICHARDSON.

I was called to the North Side to give an anesthetic for Dr. Peterson. I gave the anesthetic while the doctor drilled into five anterior teeth which had been dwarfed from infancy. He removed the live pulps, put in temporary stopping, and then I took my forceps and extracted seven teeth. The whole operation lasted about twelve minutes. It was absolutely painless. The young lady recovered promptly when I turned off the gas, and reported that she had a pleasant time at school.

Not long ago I gave gas for Dr. Morgenthau, a prominent physician of this city, while the doctor took the dental bur and drilled into the antrum of Highmore. I afterward extracted an upper third molar. This operation lasted about eleven minutes.

I have given gas for Dr. Crutcher, a surgeon in this city, on several occasions. Once while he amputated a finger; on two occasions while he reduced a dislocation of the shoulder joint, carrying the patient to the stage of muscular relaxation, so that he was enabled to reduce the dislocated shoulder. These operations lasted, I think, about eight minutes. On another occasion I gave gas for Dr. Keefe, who drilled above the cuspid tooth and curetted a lot of necrosed tissue. The operation lasted eight or nine minutes, and was a perfect success.

Two weeks ago I was present and took general charge of the anesthetic for the wife of a dentist in this city, while some tubercular glands of the neck were removed. The operation lasted twenty-five minutes, and was a success. A few weeks ago Dr. Smith gave gas to an infant, three weeks old, upon whom circumcision was performed, the operation lasted fifteen minutes, with absolutely-perfect results.

The nearest failure I have ever had with the Hurd outfit during prolonged anesthesia was when I gave an anesthetic two or three months ago for Dr. Bentley. He opened over the central, lateral and cuspid teeth, for the purpose of draining a large pus pocket. I put the patient asleep and the anesthetic worked nicely for about seven minutes' but during the latter part of the operation the patient seemed to feel pain. I was able to keep him under the influence of the anesthetic for five minutes more, until Dr. Bentley completed the operation, but the patient told us that he felt considerable pain during the latter part of the operation. That is the nearest to a partial failure I have ever had with this outfit. But I have had some complete failures. I have been utterly unable to put some patients to sleep. A year or so ago a lady came into my office with this history: Dr. Coulter, a specialist in New York City, had given her gas twice, without any effect, and her dentist recommended her to come to me. She was as good a patient as ever sat down in my chair. At first I used the Hurd outfit, using about forty gallons of gas, without any effect. I then used the old outfit, and gave her about as much more, with the same result. I might as well have given her fresh air. I dismissed her for that day, and repeated the anesthesia the next day, with no effect. She gave up, and on another occasion she took gas without any trouble whatever, and had four or five teeth extracted. I do not know how to account for that.

I once gave a boy fifty gallons of gas, using both outfits, with no effect. I dismissed him, and made an appointment for the next day. He came; I gave him gas, and never had a patient go to sleep so easily in my life. This must have been due to a difference in the physical condition of the boy, and yet there was apparently no difference. He did not struggle against taking the gas. These are things I have never been able to explain, and I do not know how to account for them. I mention them as peculiar cases.

I do not think that any gas outfit will ever be used extensively for major operations. In minor dental and surgical operations these outfits ought to be used a great deal more than they now are. When one has but thirty seconds in which to operate, he must necessarily be a very skillful operator.

I use chloroform with gas, or vitalized air, so-called, but I do not think it is necessary. I do believe that chloroform saves gas and makes the anesthesia a little more profound. In a clinic last winter at the alumni association meeting at the Northwestern University Dental School, I operated on forty-two cases that afternoon, without a failure. It simply worked perfectly. There was not a patient who made a sound or move. I was under the impression that I was using chloroform with the gas during that entire clinic. I was afterward informed that my assistant had spilled the chloroform, and I had given the entire clinic without it. During an administration of gas, lasting some three minutes, a patient will not absorb more than two drops of the chloroform, and about ten drops is all I have ever put in my cup for one day.

About two weeks ago Dr. Hewitt and I gave a clinic at the Chicago College of Dental Surgery. He gave chloroform, and I extracted the teeth. I took out some sixty-five or seventy teeth that morning for eight patients. I removed fourteen teeth for one lady patient. There was not a patient who felt any pain. They all knew that their teeth were being extracted, but there was no pain. I do not think the dental profession makes use of Dr. Hewitt's chloroform method as often as it should. I have used it a number of times. Occasionally I have a patient who is accustomed to taking chloroform, and will not have anything but chloroform as an anesthetic. In such a case I have given chloroform after the method of Dr. Hewitt, and in every case it has worked satisfactorily. I think the dental profession is needlessly alarmed about the use of chloroform.

Dr. J. W. Slonaker. With pure nitrous oxid, made from pure nitrate of ammonia, and made in your office by some one competent to make it (and much depends on the manufacture of the gas), I can produce anesthesia more quickly and keep my patients under the effect of the gas long enough for ninety-nine percent of the operations I have to perform. I have so few unpleasant effects, such as sickness, etc., that it practically amounts to nothing. When I wish to prolong the anesthetic effect, I carry my patients thoroughly under the influence of the gas; then admit a small amount of air, allowing them to partially recover consciousness; then I give them the gas full strength, carrying them as far as judicious. By so doing I am enabled to perform more extensive operations.

The administration of nitrous oxid to persons affected with heart trouble is admissible, provided the person's general health is good. To those with kidney disease, nervous prostration, and general nervous disorders, I find no difficulty. There is only one class of persons to whom I refuse to administer gas. I refer to those who have had a stroke of apoplexy, and should consider one criminally liable who should knowingly give it under those conditions, because the action of nitrous oxid dilates the brain and it would protrude from an opening made in the skull, showing that a rupture might easily occur.

In cases of pregnancy I consider it perfectly safe.

Persons having a drug habit, or inebriates, should be handled with great care.

I want to say a few words in relation to the administration of nitrous oxid for prolonged minor surgical operations. My experience extends over some twenty-seven or twenty-eight years, and in that time I have given nitrous oxide as long as ten minutes. I assisted in one case for an amputation of the femur, in another case for amputation of the foot, and frequently in other cases, as the amputation of fingers and toes, also numerous other small or minor surgical operations.

There is a theory that I have been thinking of for a number of years. It is only a theory, because I have been unable to find any one who could give me accurate statistics relative to the operation of morphin on the brain. We know that morphin, opium, etc., contracts the pupil of the eye. Now, those patients who are addicted to that habit are the most difficult to whom to administer nitrous oxid, and I have reasoned it out in this way, that the operation of morphin on the brain contracts the coats of the arteries and veins of the brain. I am speaking of those who use it continuously, taking certain quantities every day. The operation of nitrous oxide dilates the brain tissue, allowing, as I said a few minutes ago, the brain to enlarge to fill the entire skull cavity. Now, when a patient comes to you who is addicted to the use of morphine, that being, of course, my theory, the brain is abnormally contracted. Those patients will take all the way from twice to three times as much gas, and still remain conscious, but as quick as the wink of the eye they lose consciousness, and the symptoms produced are of such a character that they are really alarming. Given two or three breaths of air, they are conscious again, or semi-conscious, so much so that it is utterly impossible to do any more than to extract, say, three or four teeth, after which you have terrific screaming, fighting and kicking—crazy actions. In the cases of those who are addicted to the use of liquor (I am not speaking of men who take a drink before they come to your office, but men who drink from ten to fifteen drinks of whisky a day), their brain is enlarged. There is undoubtedly an inflammation of the brain. You administer gas to such men, and very few breaths of pure gas are sufficient to place them in an unconscious condition. Still they recover quickly from the influence of the gas, because they have had so little of it, and consequently you have very little time to do anything for them. In speaking of that, I am reminded of a case that came under my observation some years ago, one that I saw with Dr. Thomas, of Philadelphia. The patient was a man who had taken a great deal of liquor. He sat down in the chair, and absolutely did not take one breath of gas, but held his breath for something like thirty seconds, after which he lost complete consciousness from over-stimulation. His teeth were extracted, and he told us that he thought nitrous oxid gas was the most glorious anesthetic that he had ever taken.

I pay no attention to motion of fingers in contouring, but I do to the breathing and appearance of the patient, and everything that goes with the administration of nitrous oxid. The cyanotic condition has nothing to do with telling whether the patient is unconscious or not. Frequently in anesthetizing patients, indications of cyanosis are hardly noticeable. I want to see the condition of the lips. I want to see how the patient is breathing. I guard against any noise in the room during anesthesia. If there is, it will make it impossible to hear, and quite often we have patients who are old and delicate, who breathe so slowly and quietly that you can hardly hear them do so. In administering nitrous oxid the effect is cumulative. The patient can breathe, say, a few breaths of nitrous oxid and be perfectly conscious. You can take the inhaler away from the patient, and in fifteen seconds or so the patient is unconscious. If I have a large man to anesthetize, I treat him as I did one this morning, one weighing 275 pounds—plethoric. I gave him gas, and when I removed the inhaler he was conscious. I waited for about ten seconds. He lost consciousness. I extracted his teeth. He came to, and did not know anything about it. If I had given gas until he was profoundly under its influence, I would have made too marked an impression upon his nervous system. It is largely a question of experience, and as for extracting ten or fifteen teeth, the greatest number of teeth extracted by me during one administration of nitrous oxid is twenty-seven. I frequently extract nineteen or twenty, so that I have not found it necessary to use any other method. Of course, occasionally we have peculiar patients, as Dr. Richardson says. We have patients who are difficult to anesthetize. However, I have never seen a patient, unless it was one addicted to the morphin or opium habit, that I could not put under the influence of nitrous oxid profoundly.—*Review.*

